# Early-Stage HCC Percutaneous Locoregional Management: East versus West Perspectives

**DOI:** 10.3390/cancers15153988

**Published:** 2023-08-06

**Authors:** Roberto Iezzi, Alessandro Posa, Andrea Contegiacomo, In Joon Lee, Reto Bale, Alessandro Tanzilli, Lorenzo Tenore, Felice Giuliante, Antonio Gasbarrini, Shraga Nahum Goldberg, Tobias Jakobs, Maurizio Pompili, Irene Bargellini, Evis Sala, Hyo-Cheol Kim

**Affiliations:** 1 Department of Diagnostic Imaging, Oncologic Radiotherapy and Hematology, A. Gemelli University Hospital Foundation IRCCS, 00168 Rome, Italy; alessandro.posa@policlinicogemelli.it (A.P.); andrea.contegiacomo@policlinicogemelli.it (A.C.); lorenzo.tenore01@icatt.it (L.T.); evis.sala@policlinicogemelli.it (E.S.); 2 School of Medicine, Catholic University of the Sacred Heart, 00168 Rome, Italy; felice.giuliante@unicatt.it (F.G.); antonio.gasbarrini@policlinicogemelli.it (A.G.); maurizio.pompili@policlinicogemelli.it (M.P.); 3 Department of Radiology, National Cancer Center, Goyang 10408, Republic of Korea; 2injoon@gmail.com; 4 Section of Interventional Oncology-Microinvasive Therapy (SIP), Department of Radiology, Medical University of Innsbruck, 6020 Innsbruck, Austria; reto.bale@i-med.ac.at; 5 Department of Radiology, Santa Maria Goretti Hospital, 04100 Latina, Italy; a.tanzilli@ausl.latina.it; 6 Hepatobiliary Surgery Unit, A. Gemelli University Hospital Foundation IRCCS, 00168 Rome, Italy; 7 Internal Medicine and Gastroenterology Unit, A. Gemelli University Hospital Foundation IRCCS, 00168 Rome, Italy; 8 Division of Image-Guided Therapy, Department of Radiology, Hadassah Hebrew University Medical Center, Jerusalem 91120, Israel; sgoldber@bidmc.harvard.edu; 9 Clinic of Interventional Radiology, Hospital Barmherzige Brueder, 93049 Munich, Germany; tobias.jakobs@barmherzige-muenchen.de; 10 Institute of Radiology, Candiolo Hospital, 10126 Turin, Italy; segreteria.rx@ircc.it; 11 Department of Radiology, Seoul National University Hospital, Seoul National University College of Medicine, Seoul 03080, Republic of Korea; radioembolization@snu.ac.kr

**Keywords:** hepatocellular carcinoma, interventional oncology, ablation, chemoembolization, locoregional treatments, radioembolization

## Abstract

**Simple Summary:**

The aim of this review is to evaluate the different approaches to early-stage hepatocellular carcinoma according to Western and Eastern interventional radiology perspectives based on the most recent literature. Understanding different perspectives and guidelines can help in selecting the correct indication of locoregional procedures (mostly ablation, chemoembolization, and radioembolization) for every patient.

**Abstract:**

Hepatocellular carcinoma represents an important cause of death worldwide. Early-stage hepatocellular carcinoma patients not suitable for surgery can be treated with a variety of minimally invasive locoregional interventional oncology techniques. Various guidelines in different countries address the treatment of hepatocellular carcinoma, but the actual treatment is usually discussed by a multidisciplinary tumor board in a personalized manner, leading to potential treatment differences based on Western and Eastern perspectives. The aim of this paper is to integrate literature evidence with the eminent experiences collected during a focused session at the Mediterranean Interventional Oncology (MIO) Live Congress 2023.

## 1. Introduction

Hepatocellular carcinoma (HCC) is a major cause of cancer death, with an increasing incidence [1]. Interventional oncology techniques are minimally invasive procedures, providing lower-risk alternatives to many traditional surgical therapies [2]. There are many locoregional minimally invasive treatment options, alone or in combination, that aim to improve the clinical condition of patients, particularly early-stage HCC patients [3,4,5]. Early-stage HCC can be defined, according to the Barcelona Clinic Liver Cancer (BCLC) group, as a single neoplastic nodule or up to three nodules smaller than 3 cm in size in a patient with good performance status (BCLC-A) [5]. Despite various guidelines, the actual treatment plan needs to be discussed with a local multidisciplinary tumor board with a personalized approach. This can lead to potential treatment differences based on Western and Eastern perspectives.

The aim of this paper is to integrate evidence-based literature and experience-based perceptions on locoregional treatments for early-stage HCC—collected during a focused session at the Mediterranean Interventional Oncology (MIO) Live Congress 2023—while attempting to make the information easy to access. By highlighting recent data and strategies for early-stage HCC locoregional treatment, it may assist both trainees in interventional radiology and practicing colleagues who are attempting to gain further expertise with locoregional procedures.

## 2. Local Ablation

### 2.1. Eastern Perspective

Local ablative treatments for early-stage HCC include radiofrequency ablation (RFA), microwave ablation (MWA), and cryoablation [4,5]. In terms of clinical outcome, two retrospective studies on RFA treatment of early-stage HCC with a mean lesion size of 2.2 cm (±0.8 cm) and 2.54 cm (±1.04 cm) showed 5-year overall survival of 59.7% and 60.2%, respectively [6,7]. These results are comparable to surgical resection, which achieves about 60% 5-year survival in these patients [8]. On the other side, Lee et al. evaluated the recurrence rates in 1491 BCLC-A HCC patients treated with RFA or surgery. Although the cumulative incidences of HCC recurrence decreased over time in both groups of patients, those treated with RFA had significantly higher incidences than those treated with resection; this is particularly true in HCC lesions larger than 2 cm, mostly due to the heat-sink effect, the difficult prediction of safety margins, and the difficult lesion location [9].

The Korean Liver Cancer Association (KLCA) and the National Cancer Center (NCC) guidelines recommend RFA as the treatment of choice in patients with no more than 3 nodules up to 3 cm in size [10]. Single-probe ultrasound monopolar RFA is the most common ablative treatment for HCC in the East, performed under ultrasound (US) or computed tomography (CT) guidance. Image fusion systems, such as those used to coordinate ultrasound and CT or MRI, have also been designed in recent years to increase the ability to recognize lesions, especially those smaller than 2 cm, and the accuracy of the ablation [11].

**Figure 1:**
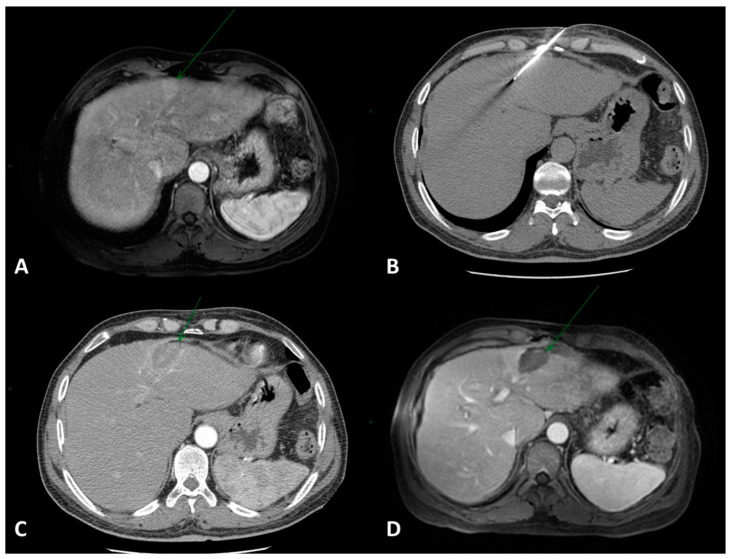
(**A**) Contrast-enhanced axial MRI showing a 2.5 × 2 cm HCC lesion in the IV hepatic segment (arrow). (**B**) CT-guided RFA was performed with one electrode. (**C**) Immediate post-procedural contrast-enhanced CT examination showing the ablation area without complications. (**D**) Post-treatment axial MRI showing complete response (arrow).

RFA yields an ablation zone of about 3 cm in diameter (Figure 1).

To achieve an adequate safety margin in tumors larger than 2 cm, multiple overlapping ablations can be required. More sophisticated RFA systems, employing multiple electrodes, improve energy delivery to obtain larger ablation volumes; Choi et al. examined the possibility of obtaining larger ablation volumes using a dual-switching probe instead of a monopolar probe without significant results; in particular, although dual-switching monopolar mode delivered higher ablation energy, it did not show better results than single-switching monopolar mode in terms of effectiveness [12].

Another technique is “no-touch” RFA, where multiple electrodes (more than 2) are placed outside the tumor margin, delivering heat to the target tumor in a centripetal manner. Hocquelet et al. retrospectively compared “no-touch” bipolar/multipolar RFA (238 patients) versus monopolar RFA (358 patients) for small HCCs and concluded that “no-touch” bipolar/multipolar RFA provides better and sustained local tumor control for small HCCs compared to monopolar RFA (treatment failure of 7.1% versus 28.7%, respectively) [13]. In a recent multicenter trial in patients with single HCC up to 2.5 cm, RFA using the “no-touch” technique obtained a 2-year local tumor progression (LTP) of 1.6%, as compared to approximately 12% after conventional RFA [14]. Another prospective randomized controlled trial by Suh et al., using twin internally cooled wet electrodes in the “no-touch” technique, obtained a 1- and 3-year cumulative incidence LTP rate of 0% in the no-touch-RFA group, compared with the 15.6% and 24.5% of conventional RFA, respectively [15].

In conclusion, RFA is a safe and effective local treatment for early-stage HCC. RFA with multiple electrodes and “no-touch” RFA improves the therapeutic efficacy of RFA [13,14].

### 2.2. Western Perspective

The toolbox of ablative interventional oncology techniques includes RFA, MWA, cryoablation, and irreversible electroporation (IRE) [16]. According to the BCLC guidelines, ablation is regarded as the first-line treatment in very early-stage HCC (BCLC-0, single lesion up to 2 cm in size, with preserved liver function and performance status (PS) equal to 0) [5].

Patients in the early BCLC stage (BCLC-A), unsuitable for surgery or transplantation, can benefit from ablative treatments; in addition, ablation can be a bridging treatment to transplantation. Ablation is considered a minimally invasive alternative for single tumors up to 3 cm in size. MWA achieves a larger ablation volume per probe. Therefore, MWA represents the preferred ablative technique for single HCCs larger than 2 cm [17,18].

A stereotactic multi-needle ablation workflow including 3D-planning, precise needle placement, and intraprocedural verification of the ablation volume by means of image fusion was developed by Bale et al. [19,20,21]. To obtain large ablation volumes and to achieve sufficient safety margins, multiple coaxial needles are placed within and around the target lesions using an optical navigation system combined with an aiming device. The coaxial needles act as spacers for the introduction of up to three RFA/MWA/IRE/cryoablation probes [19].

Bale et al. treated 188 HCCs with a median tumor size of 2.5 cm (range 1–8 cm) in 96 patients as a bridging treatment to liver transplantation using stereotactic RFA. In 183/188 nodules (93.7%), a complete histopathological response was achieved; in lesions larger than 3 cm in size, the histopathological complete response rate was 96.2% (50/52 nodules). Median overall survival (OS) was 90 months for BCLC-0 HCC (68% 5-year OS) and 65 months for BCLC-A HCC (55% 5-year OS) [22].

**Figure 2:**
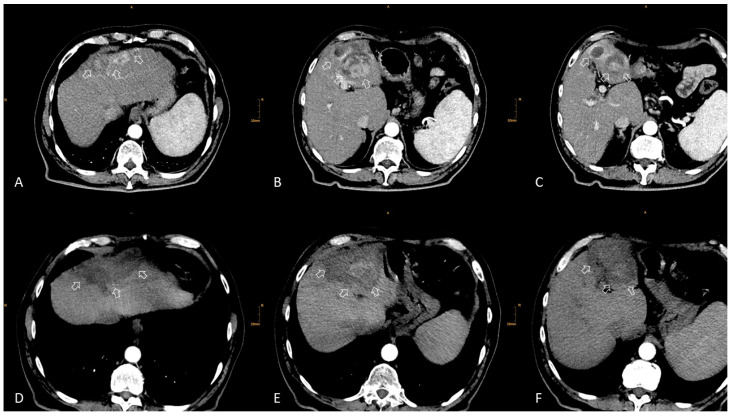
A 73-year-old male with cirrhosis and histologically verified multifocal HCC (segments II, III, Iva, and IVb) with a maximum diameter of 10.4 cm. Previous TACE treatment with a partial response. Due to portal hypertension and a hepatic venous pressure gradient (HVPG) of 10 mmHg, the patient was deemed unsuitable for surgical resection. (**A**–**C**) Pre-stereotactic RFA axial contrast-enhanced CT scans showing the large multifocal HCC (white arrows). (**D**–**F**) Post-treatment contrast-enhanced axial CT scans show a good ablation zone. 14 coaxial needles were used, for a total of 33 RFA probe positionings and an ablation time of 132 min (4 min per probe position).

In another study, 34 patients with HCC and previous surgical resection were treated by stereotactic RFA for 140 recurrent HCCs (Figure 2).

The median tumor size was 3.0 cm (range 0.5–10 cm). Local tumor recurrence was observed in 4/140 tumors (2.9%). The median OS from the date of the first stereotactic RFA was 69.1 months. The corresponding OS rates at 1, 3, and 5 years were 94.0%, 70.2%, and 53.3%, respectively [23].

Ablative treatments are not routinely recommended in BCLC-B HCCs, which usually undergo transarterial chemoembolization (TACE); however, if there are favorable conditions and the multidisciplinary tumor board team agrees, a more aggressive treatment like surgical resection or thermal ablation (the so-called “right-to-left migration strategy”) on intermediate-stage HCC patients is considered, with good results in terms of OS [24].

Adjuvant therapies in addition to percutaneous locoregional treatments can be of great help in improving the time-to-recurrence, as in the case of the anti-angiogenic chemotherapy drug Sorafenib—even though literature results are contrasting—or in the case of sartan drugs, as evidenced by Facciorusso et al. [25,26,27].

In conclusion, RFA represents the standard of care in patients with BCLC-0 and -A stage HCC and a possible alternative to surgical resection in single tumors up to 3 cm in size. Furthermore, RFA may be used as a bridging and downstaging agent before liver transplantation. MWA achieves larger ablation zones as compared to RFA and may be the preferred ablation method for lesions larger than 2 cm [5]. In addition, according to a meta-analysis by Facciorusso and colleagues, MWA seems to grant better long-term (5-year) recurrence-free survival when compared to RFA [28]. Ablation may also be applied in intermediate HCC (“right-to-left migration strategy”), based on multidisciplinary tumor board decisions. Multi-probe stereotactic-RFA with intraoperative image fusion can be used to treat neoplastic lesions with no limit in size and number (care must be taken in the case of lesions near central biliary structures or in the case of low residual liver volume) and could replace resection as a first-line local curative treatment even in lesions larger than 3 cm (“left-to-right migration”) [29].

## 3. TACE

### 3.1. Eastern Perspective

Trans-arterial therapies still represent the most commonly used approach to HCC, and conventional TACE (cTACE) is the most widely used. According to most Asian guidelines, in cases of intermediate HCC without significant vascular invasion or extrahepatic dissemination, TACE is the recommended course of therapy [30]. The main reasons are related to the possible presence of additional HCC nodules requiring diagnostic angiography (28%), difficult or complex anatomical tumor location (17%), high surgical risk (12%), patient’s refusal to surgery or ablation (10%), and risk of post-treatment liver failure [31].

Oh et al. compared curative approaches (resection and RFA) and TACE in patients with multiple small (<3 cm) HCCs within Milan criteria and found no statistical difference in long-term survival rates [32]. In contrast, another study by Lee et al. evaluated the combined use of RFA and cTACE versus exclusive treatment with cTACE in 165 patients with stage A HCC in terms of LTP and OS: the cTACE group showed higher 1-, 3-, and 5-year LTP rates than the combined group (12.5%, 31.7%, and 37.0%, respectively, in the cTACE group, vs. 7.3%, 16.5%, and 16.5%, respectively, in the combined group; *p* = 0.013). About the OS, in the cTACE group, the 1-, 3-, and 5-year OS rates were 100%, 93.2%, and 87.7%, respectively, whereas in the combination group, they were 100%, 96.6%, and 87.4%, respectively (*p* = 0.686) [33].

Treatment of HCC nodules can be performed with a segmental or superselective approach through the tumor-feeding artery. A superselective approach can optimize the therapeutic effect by reducing the damage to the surrounding non-neoplastic liver, the amount of drug administered, and the number of TACE sessions needed to induce widespread lesion necrosis [34].

Lee et al. showed that superselective subsegmental TACE can be performed using cone-beam CT (CBCT) without additional angiography in 85% of patients and in 90% of nodules [35].

The mechanism through which TACE reaches its therapeutic effect is both ischemic and cytotoxic, by infusing a Lipiodol-based emulsion (cTACE) or drug-eluting beads (DEB-TACE). DEB-TACE has better pharmacokinetic effects than cTACE without a significant difference in therapeutic outcomes. It is thought that the advantage of drug-eluting beads is that they reduce the diffusion of the drug to the periphery, improving the local anti-tumor effect of the chemotherapeutic [36].

A prospective multicenter study by Lee et al. on DEB-TACE found that the median progression-free survival (PFS) was 9.3 months; in a “per-lesion” analysis, nodules between 2 and 5 cm got better results than those smaller than 2 cm [34]. A retrospective study comparing superselective DEB-TACE and cTACE showed that cTACE had significantly better PFS in lesions smaller than 3 cm, whereas local DEB-TACE tumor control was better in lesions ranging from 2.1 to 5 cm [37]. A known problem of cTACE is the refractoriness of patients with HCC after multiple treatments. Li et al. retrospectively evaluated progression-free survival (PFS) in 81 patients with a history of previous cTACE treatments who underwent a subsequent cTACE or DEB-TACE session; they found a better objective response rate, PFS rate, and OS rate in the group undergoing DEB-TACE (respectively *p* = 0.003, *p* = 0.028, and *p* = 0.037) [38].

### 3.2. Western Perspective

The 2009 PRECISION-V randomized study stated that Child-Pugh class-B HCC patients with reduced performance status, as well as patients with bilobar HCC, would benefit from DEB-TACE both clinically and at imaging follow-up [39]. These results of DEB-TACE were confirmed by various Authors, with more than 41% 4-year OS, greater than cTACE [37,40].

In summary, DEB-TACE is non-inferior to cTACE in terms of patient survival, has fewer side effects, and grants a shorter hospital stay compared to cTACE [41].

**Figure 3:**
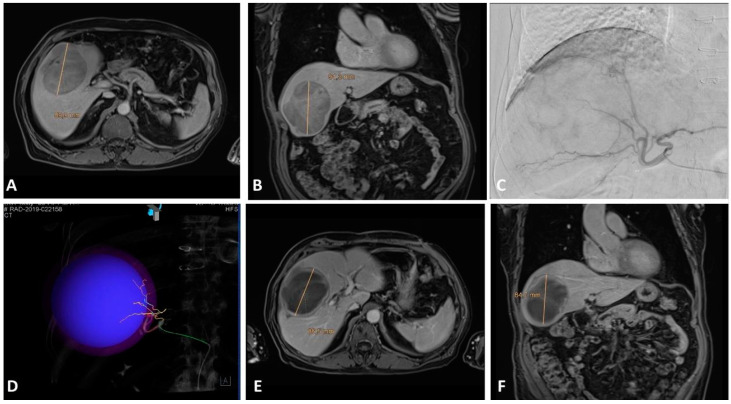
(**A**,**B**) Axial and coronal contrast-enhanced MRI images showing a 9 cm dishomogenously hypointense lesion in the right hepatic lobe in a BCLC-B patient. (**C**) Pre-treatment diagnostic angiography showing tumor-feeding arteries. (**D**) CBCT coronal VR reconstruction showing target volume and color-coded tumor-feeding arteries. (**E**,**F**) Axial and coronal contrast-enhanced MRI images showing 3-month post-DEB-TACE results with great tumor necrosis.

Nowadays, TACE is still considered a standard of care in intermediate stage (BCLC-B) HCC patients (Figure 3) and represents a bridging therapy in patients on the liver transplant list as well as a valuable downstaging tool; TACE could also be performed in early stage (BCLC-A) HCC patients deemed not eligible for ablation, resection, or transplantation, as well as in selected advanced stage (BCLC-C) patients—despite guidelines [42,43,44,45].

Going beyond guidelines, TACE can be combined with systemic therapies, as demonstrated by a phase II multicenter randomized trial from Japan on the combination of TACE plus Sorafenib (a tyrosine kinase inhibitor) versus TACE alone in patients with HCC, which showed a significant increase in PFS rates (even though without an increase in OS rates) [46].

The combination of TACE and immunotherapy represents a promising research area: immunotherapy can increase the effects of locoregional therapy in HCC, as locoregional therapies produce an immune response that can be augmented via immune checkpoint inhibition [47].

## 4. TARE

### 4.1. Eastern Perspective

TARE has excellent outcomes for patients in the BCLC-A stage. However, the indication is often limited due to high costs. It can be used in cases of unresectable lesions, heavy comorbidities, or a patient’s rejection of surgery [48].

Asian guidelines concerning TARE are less standardized than Western ones [49].

Yim et al. have assessed the OS (18.6 months) and tumor response (45%, 40% in BCLC C) in 149 patients with different HCC stages who underwent TARE, with results overlapping with those of similar Western studies [50].

Radiation segmentectomy works very well for lesions smaller than 8 cm in peripheral locations, using high radiation activity with low disease recurrence rates. TARE may replace portal vein embolization (PVE), as it stimulates healthy liver growth; moreover, it offers the possibility to control the tumor during transplant-list waiting time, but with a long hypertrophy time (about 3 months) [51].

### 4.2. Western Perspective

TARE is an effective treatment for early-stage HCC, with a long-lasting complete response and high overall survival. According to the 2022 BCLC criteria, TARE can be performed on patients with single HCCs up to 8 cm in size [5]. In the LEGACY study by Salem et al., 162 patients with single HCCs up to 8 cm in size were treated with radioembolization [52]. Even though the majority of the patients had a nodule smaller than 3 cm in size and the responses were not stratified according to tumor size, the results were interesting, with a high objective response rate (88%) and similar OS rates between TARE and surgical resection. According to Salem et al., radiation segmentectomy can achieve 100% complete pathological necrosis. Other studies showed that segmental TARE is better than segmental TACE at achieving a complete and durable response [53].

**Figure 4:**
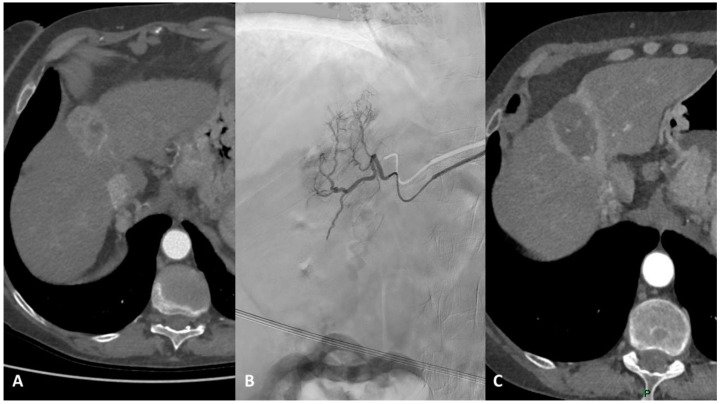
(**A**) Contrast-enhanced axial CT examination showing a 5 cm HCC lesion in the IV hepatic segment. (**B**) Angiography examination showing tumor staining of the HCC lesion prior to TARE. (**C**) Post-treatment contrast-enhanced axial CT examination showing the complete necrosis of the HCC lesion.

Beyond guidelines, in cases of solitary unresectable HCC suitable for TARE, it is necessary to classify the patients based on tumor size to choose the best treatment, also considering the costs of each treatment and the corresponding result: for lesions smaller than 3 cm, ablation represents the first option; for lesions between 3 and 5 cm, literature supports the use of surgery or combined treatment (TACE plus ablation), whereas TARE represents an effective therapeutic option for unresectable single lesions larger than 5 cm. Both TACE and TARE can be considered bridge or downstaging therapies for liver transplantation (Figure 4) [54].

In the case of multifocal early-stage HCC, it is important to consider lesion distribution: TARE is effective if the disease is spread in one or two contiguous segments. In the case of lesions in non-adjacent segments or in different lobes, TACE or ablation (or a combination of both) should be preferred.

TARE’s disadvantages are its higher costs and procedural complexity compared to other locoregional procedures [55]. Many efforts have been made to reduce the costs and complexity of TARE, for example, by skipping the macroaggregated albumin (MAA) scan and using CBCT as a surrogate to assess selective tumor uptake and exclude extrahepatic uptake [56].

## 5. Conclusions

The best approach for patients with early HCC is still debatable as it is based on tumor characteristics, liver function, performance status, patient comorbidities, center and operator skills and experience, as well as device availability. Innovation in diagnostic and surveillance technology, improvements in locoregional treatment modalities, the application of combined therapies, and expanded indications for standard procedures hold promise for improving outcomes for HCC. Treatment selection can also be influenced by cultural and socioeconomic differences, with different Western and Eastern perspectives.

In the 2022 BCLC strategy, the expert clinical decision-making approach is the only one formally permitting tailored treatment based on not only patient and tumor characteristics but also local expertise and technical availability. A personalized multidisciplinary discussion for every single patient and every single HCC lesion can help in finding the most suitable treatment for each case [5].

## References

[1] Chidambaranathan-Reghupaty S., Fisher P.B., Sarkar D. (2021). Hepatocellular carcinoma (HCC): Epidemiology, etiology and molecular classification. Adv. Cancer Res..

[2] Makary M.S., Ramsell S., Miller E., Beal E.W., Dowell J.D. (2021). Hepatocellular carcinoma locoregional therapies: Outcomes and future horizons. World J. Gastroenterol..

[3] Gnutzmann D., Kortes N., Sumkauskaite M., Schmitz A., Weiss K.H., Radeleff B. (2018). Transvascular therapy of Hepatocellular Carcinoma (HCC), status and developments. Minim. Invasive Ther. Allied Technol..

[4] Lencioni R., de Baere T., Martin R.C., Nutting C.W., Narayanan G. (2015). Image-Guided Ablation of Malignant Liver Tumors: Recommendations for Clinical Validation of Novel Thermal and Non-Thermal Technologies—A Western Perspective. Liver Cancer.

[5] Reig M., Forner A., Rimola J., Ferrer-Fàbrega J., Burrel M., Garcia-Criado Á., Kelley R.K., Galle P.R., Mazzaferro V., Salem R. (2022). BCLC strategy for prognosis prediction and treatment recommendation: The 2022 update. J. Hepatol..

[6] Kim Y.S., Lim H.K., Rhim H., Lee M.W., Choi D., Lee W.J., Paik S.W., Koh K.C., Lee J.H., Choi M.S. (2013). Ten-year outcomes of percutaneous radiofrequency ablation as first-line therapy of early hepatocellular carcinoma: Analysis of prognostic factors. J. Hepatol..

[7] Shiina S., Tateishi R., Arano T., Uchino K., Enooku K., Nakagawa H., Asaoka Y., Sato T., Masuzaki R., Kondo Y. (2012). Radiofrequency ablation for hepatocellular carcinoma: 10-year outcome and prognostic factors. Am. J. Gastroenterol..

[8] Ju M.R., Yopp A.C. (2021). Surgical resection of early stage hepatocellular carcinoma: Balancing tumor biology with the host liver. Chin. Clin. Oncol..

[9] Lee H.A., Lee Y.S., Kim B.K., Jung Y.K., Kim S.U., Park J.Y., Kim J.H., An H., Kim D.Y., Yim H.J. (2021). Change in the Recurrence Pattern and Predictors over Time after Complete Cure of Hepatocellular Carcinoma. Gut Liver.

[10] Korean Liver Cancer Association (KLCA), National Cancer Center (NCC) Korea (2022). 2022 KLCA-NCC Korea Practice Guidelines for the Management of Hepatocellular Carcinoma. Korean J. Radiol..

[11] Lee D.H., Lee J.M. (2018). Recent Advances in the Image-Guided Tumor Ablation of Liver Malignancies: Radiofrequency Ablation with Multiple Electrodes, Real-Time Multimodality Fusion Imaging, and New Energy Sources. Korean J. Radiol..

[12] Choi J.W., Lee J.M., Lee D.H., Yoon J.H., Kim Y.J., Lee J.H., Yu S.J., Cho E.J. (2021). Radiofrequency Ablation Using a Separable Clustered Electrode for the Treatment of Hepatocellular Carcinomas: A Randomized Controlled Trial of a Dual-Switching Monopolar Mode Versus a Single-Switching Monopolar Mode. Korean J. Radiol..

[13] Hocquelet A., Aubé C., Rode A., Cartier V., Sutter O., Manichon A.F., Boursier J., N’kontchou G., Merle P., Blanc J.F. (2017). Comparison of no-touch multi-bipolar vs. monopolar radiofrequency ablation for small HCC. J. Hepatol..

[14] Lee D.H., Lee M.W., Kim P.N., Lee Y.J., Park H.S., Lee J.M. (2021). Outcome of No-Touch Radiofrequency Ablation for Small Hepatocellular Carcinoma: A Multicenter Clinical Trial. Radiology.

[15] Suh Y.S., Choi J.W., Yoon J.H., Lee D.H., Kim Y.J., Lee J.H., Yu S.J., Cho E.J., Yoon J.H., Lee J.M. (2021). No-Touch vs. Conventional Radiofrequency Ablation Using Twin Internally Cooled Wet Electrodes for Small Hepatocellular Carcinomas: A Randomized Prospective Comparative Study. Korean J. Radiol..

[16] Öcal O., Rössler D., Ricke J., Seidensticker M. (2022). Advances in Diagnostic and Interventional Radiology in Hepatocellular Carcinoma. Dig. Dis..

[17] Vietti Violi N., Duran R., Guiu B., Cercueil J.P., Aubé C., Digklia A., Pache I., Deltenre P., Knebel J.F., Denys A. (2022). Efficacy of microwave ablation versus radiofrequency ablation for the treatment of hepatocellular carcinoma in patients with chronic liver disease: A randomised controlled phase 2 trial. Lancet Gastroenterol. Hepatol..

[18] Han J., Fan Y.C., Wang K. (2020). Radiofrequency ablation versus microwave ablation for early stage hepatocellular carcinoma: A PRISMA-compliant systematic review and meta-analysis. Medicine.

[19] Bale R., Widmann G., Haidu M. (2011). Stereotactic radiofrequency ablation. Cardiovasc. Intervent Radiol..

[20] Schullian P., Johnston E.W., Putzer D., Eberle G., Laimer G., Bale R. (2020). Safety and efficacy of stereotactic radiofrequency ablation for very large (≥8 cm) primary and metastatic liver tumors. Sci. Rep..

[21] Schullian P., Putzer D., Eberle G., Laimer G., Bale R. (2020). Simultaneous Stereotactic Radiofrequency Ablation of Multiple (≥4) Liver Tumors: Feasibility, Safety, and Efficacy. J. Vasc. Interv. Radiol..

[22] Bale R., Schullian P., Eberle G., Putzer D., Zoller H., Schneeberger S., Manzl C., Moser P., Oberhuber G. (2019). Stereotactic Radiofrequency Ablation of Hepatocellular Carcinoma: A Histopathological Study in Explanted Livers. Hepatology.

[23] Schullian P., Laimer G., Putzer D., Levy E., Braunwarth E., Stättner S., Bale R. (2020). Stereotactic radiofrequency ablation as first-line treatment of recurrent HCC following hepatic resection. Eur. J. Surg. Oncol..

[24] Mahnken A.H. (2022). Leitliniengerechte Anwendung der Thermoablation beim hepatozellulären Karzinom [Guideline-based thermal ablation of hepatocellular carcinoma]. Radiologe.

[25] Zhou Q., Wang X., Li R., Wang C., Wang J., Xie X., Li Y., Li S., Mao X., Liang P. (2022). Sorafenib as adjuvant therapy following radiofrequency ablation for recurrent hepatocellular carcinoma within Milan criteria: A multicenter analysis. J. Gastroenterol..

[26] Seidensticker M., Öcal O., Schütte K., Malfertheiner P., Berg T., Loewe C., Klümpen H.J., van Delden O., Ümütlü M.R., Ben Khaled N. (2023). Impact of adjuvant sorafenib treatment after local ablation for HCC in the phase II SORAMIC trial. JHEP Rep..

[27] Facciorusso A., Abd El Aziz M.A., Cincione I., Cea U.V., Germini A., Granieri S., Cotsoglou C., Sacco R. (2020). Angiotensin Receptor 1 Blockers Prolong Time to Recurrence after Radiofrequency Ablation in Hepatocellular Carcinoma patients: A Retrospective Study. Biomedicines.

[28] Facciorusso A., Abd El Aziz M.A., Tartaglia N., Ramai D., Mohan B.P., Cotsoglou C., Pusceddu S., Giacomelli L., Ambrosi A., Sacco R. (2020). Microwave Ablation Versus Radiofrequency Ablation for Treatment of Hepatocellular Carcinoma: A Meta-Analysis of Randomized Controlled Trials. Cancers.

[29] Zhu F., Rhim H. (2019). Thermal ablation for hepatocellular carcinoma: What’s new in 2019. Chin. Clin. Oncol..

[30] Cho Y., Kim B.H., Park J.W. (2023). Overview of Asian clinical practice guidelines for the management of hepatocellular carcinoma: An Asian perspective comparison. Clin. Mol. Hepatol..

[31] Chang Y., Jeong S.W., Young Jang J., Jae Kim Y. (2020). Recent Updates of Transarterial Chemoembolilzation in Hepatocellular Carcinoma. Int. J. Mol. Sci..

[32] Oh J.H., Sinn D.H., Choi G.S., Kim J.M., Joh J.W., Kang T.W., Hyun D., Kang W., Gwak G.Y., Paik Y.H. (2020). Comparison of outcome between liver resection, radiofrequency ablation, and transarterial therapy for multiple small hepatocellular carcinoma within the Milan criteria. Ann. Surg. Treat. Res..

[33] Lee H., Yoon C.J., Seong N.J., Jeong S.H., Kim J.W. (2018). Comparison of Combined Therapy Using Conventional Chemoembolization and Radiofrequency Ablation Versus Conventional Chemoembolization for Ultrasound-Invisible Early-Stage Hepatocellular Carcinoma (Barcelona Clinic Liver Cancer Stage 0 or A). Korean J. Radiol..

[34] Lee I.J., Lee J.H., Lee Y.B., Kim Y.J., Yoon J.H., Yin Y.H., Lee M., Hur S., Kim H.C., Jae H.J. (2019). Effectiveness of drug-eluting bead transarterial chemoembolization versus conventional transarterial chemoembolization for small hepatocellular carcinoma in Child-Pugh class A patients. Ther. Adv. Med. Oncol..

[35] Lee I.J., Chung J.W., Yin Y.H., Kim H.C., Kim Y.I., Jae H.J., Park H.J. (2015). Cone-Beam Computed Tomography (CBCT) Hepatic Arteriography in Chemoembolization for Hepatocellular Carcinoma: Performance Depicting Tumors and Tumor Feeders. Cardiovasc. Intervent Radiol..

[36] Bzeizi K.I., Arabi M., Jamshidi N., Albenmousa A., Sanai F.M., Al-Hamoudi W., Alghamdi S., Broering D., Alqahtani S.A. (2021). Conventional Transarterial Chemoembolization Versus Drug-Eluting Beads in Patients with Hepatocellular Carcinoma: A Systematic Review and Meta-Analysis. Cancers.

[37] Song J.E., Kim D.Y. (2017). Conventional vs. drug-eluting beads transarterial chemoembolization for hepatocellular carcinoma. World J. Hepatol..

[38] Li H., Wu F., Duan M., Zhang G. (2019). Drug-eluting bead transarterial chemoembolization (TACE) vs. conventional TACE in treating hepatocellular carcinoma patients with multiple conventional TACE treatments history: A comparison of efficacy and safety. Medicine.

[39] Lammer J., Malagari K., Vogl T., Pilleul F., Denys A., Watkinson A., Pitton M., Sergent G., Pfammatter T., Terraz S. (2010). Prospective randomized study of doxorubicin-eluting-bead embolization in the treatment of hepatocellular carcinoma: Results of the PRECISION V study. Cardiovasc. Intervent Radiol..

[40] Golfieri R., Giampalma E., Renzulli M., Cioni R., Bargellini I., Bartolozzi C., Breatta A.D., Gandini G., Nani R., Gasparini D. (2014). Randomised controlled trial of doxorubicin-eluting beads vs. conventional chemoembolisation for hepatocellular carcinoma. Br. J. Cancer.

[41] Burrel M., Reig M., Forner A., Barrufet M., de Lope C.R., Tremosini S., Ayuso C., Llovet J.M., Real M.I., Bruix J. (2012). Survival of patients with hepatocellular carcinoma treated by transarterial chemoembolisation (TACE) using Drug Eluting Beads. Implications for clinical practice and trial design. J. Hepatol..

[42] Byrne T.J., Rakela J. (2016). Loco-regional therapies for patients with hepatocellular carcinoma awaiting liver transplantation: Selecting an optimal therapy. World J. Transplant..

[43] Chapman W.C., Majella Doyle M.B., Stuart J.E., Vachharajani N., Crippin J.S., Anderson C.D., Lowell J.A., Shenoy S., Darcy M.D., Brown D.B. (2008). Outcomes of neoadjuvant transarterial chemoembolization to downstage hepatocellular carcinoma before liver transplantation. Ann. Surg..

[44] Affonso B.B., Galastri F.L., da Motta Leal Filho J.M., Nasser F., Falsarella P.M., Cavalcante R.N., de Almeida M.D., Felga G.E.G., Valle L.G.M., Wolosker N. (2019). Long-term outcomes of hepatocellular carcinoma that underwent chemoembolization for bridging or downstaging. World J. Gastroenterol..

[45] Luo J., Guo R.P., Lai E.C., Zhang Y.J., Lau W.Y., Chen M.S., Shi M. (2011). Transarterial chemoembolization for unresectable hepatocellular carcinoma with portal vein tumor thrombosis: A prospective comparative study. Ann. Surg. Oncol..

[46] Kudo M., Ueshima K., Ikeda M., Torimura T., Tanabe N., Aikata H., Izumi N., Yamasaki T., Nojiri S., Hino K. (2020). Randomised, multicentre prospective trial of transarterial chemoembolisation (TACE) plus sorafenib as compared with TACE alone in patients with hepatocellular carcinoma: TACTICS trial. Gut.

[47] Finn R.S., Qin S., Ikeda M., Galle P.R., Ducreux M., Kim T.Y., Kudo M., Breder V., Merle P., Kaseb A.O. (2020). Atezolizumab plus Bevacizumab in Unresectable Hepatocellular Carcinoma. N. Engl. J. Med..

[48] Miller F.H., Lopes Vendrami C., Gabr A., Horowitz J.M., Kelahan L.C., Riaz A., Salem R., Lewandowski R.J. (2021). Evolution of Radioembolization in Treatment of Hepatocellular Carcinoma: A Pictorial Review. Radiographics.

[49] Torimura T., Iwamoto H. (2022). Treatment and the prognosis of hepatocellular carcinoma in Asia. Liver Int..

[50] Yim S.Y., Chun H.S., Lee J.S., Lim J.H., Kim T.H., Kim B.K., Kim S.U., Park J.Y., Ahn S.H., Kim G.M. (2022). Transarterial Radioembolization for Unresectable Hepatocellular Carcinoma: Real-Life Efficacy and Safety Analysis of Korean Patients. Cancers.

[51] Liebl M., Pedersoli F., Zimmermann M., Schulze-Hagen M., Truhn D., Sieben P., von Stillfried S., Tschinaev A., Heinzel A., Kuhl C.K. (2021). Induction of Contralateral Hepatic Hypertrophy by Unilobar Yttrium-90 Transarterial Radioembolization versus Portal Vein Embolization: An Animal Study. J. Vasc. Interv. Radiol..

[52] Salem R., Johnson G.E., Kim E., Riaz A., Bishay V., Boucher E., Fowers K., Lewandowski R., Padia S.A. (2021). Yttrium-90 Radioembolization for the Treatment of Solitary, Unresectable HCC: The LEGACY Study. Hepatology.

[53] Padia S.A., Kwan S.W., Roudsari B., Monsky W.L., Coveler A., Harris W.P. (2014). Superselective yttrium-90 radioembolization for hepatocellular carcinoma yields high response rates with minimal toxicity. J. Vasc. Interv. Radiol..

[54] Lopez-Lopez V., Miura K., Kuemmerli C., Capel A., Eshmuminov D., Ferreras D., Baroja-Mazo A., Cascales-Campos P., Jiménez-Mascuñán M.I., Pons J.A. (2023). Selecting the Appropriate Downstaging and Bridging Therapies for Hepatocellular Carcinoma: What Is the Role of Transarterial Radioembolization? A Pooled Analysis. Cancers.

[55] Ljuboja D., Ahmed M., Ali A., Perez E., Subrize M.W., Kaplan R.S., Sarwar A. (2021). Time-Driven Activity-Based Costing in Interventional Oncology: Cost Measurement and Cost Variability for Hepatocellular Carcinoma Therapies. J. Am. Coll. Radiol..

[56] Gabr A., Ranganathan S., Mouli S.K., Riaz A., Gates V.L., Kulik L., Ganger D., Maddur H., Moore C., Hohlastos E. (2020). Streamlining radioembolization in UNOS T1/T2 hepatocellular carcinoma by eliminating lung shunt estimation. J. Hepatol..

